# Long-Lasting Effect after Single Hyaluronate Injection for Unilateral Vocal Fold Paralysis: Does Concentration Matter?

**DOI:** 10.3390/biom11111580

**Published:** 2021-10-26

**Authors:** Yi-Chieh Lee, Yu-Cheng Pei, Yi-An Lu, Hsiu-Feng Chung, Hsueh-Yu Li, Li-Ang Lee, Tuan-Jen Fang

**Affiliations:** 1 Department of Otolaryngology Head & Neck Surgery, Chang Gung Memorial Hospital at Linkou, 5 Fushing St., Taoyuan 333, Taiwan; yichiehli@gmail.com (Y.-C.L.); b9402009@cgmh.org.tw (Y.-A.L.); s77001birdphone@cgmh.org.tw (H.-F.C.); hyli38@adm.cgmh.org.tw (H.-Y.L.); 5738@cgmh.org.tw (L.-A.L.); 2 London School of Hygiene and Tropical Medicine, London WC1E 7HT, UK; 3 School of Medicine, Chang Gung University, 259 Wen-Hwa 1st Road, Taoyuan 333, Taiwan; yspeii@gmail.com; 4 Department of Physical Medicine and Rehabilitation, Chang Gung Memorial Hospital at Linkou and Taoyuan, 5 Fushing St., Taoyuan 333, Taiwan; 5 Center for Vascularized Composite Allotransplantation, Chang Gung Memorial Hospital, 5 Fushing St., Taoyuan 333, Taiwan; 6 Healthy Ageing Research Center, Chang Gung University, 259 Wen-Hwa 1st Road, Taoyuan 333, Taiwan

**Keywords:** hyaluronate injection, HA, unilateral vocal fold paralysis

## Abstract

Background: Early injection laryngoplasty (EIL) using hyaluronic acid (HA) is an effective treatment for glottic insufficiency in patients with acute unilateral vocal fold paralysis (UVFP). Most patients benefit by showing improvement in voice and quality of life and implied reduced need for permanent laryngoplasty. However, injected HA might resolve within a short period, so its long-term outcomes and the need for secondary procedures need to be clarified. Methods: Patients who underwent EIL with HA for acute UVFP from January 2015 to December 2018 were included. The factors that may associate with the prognosis including voice performance and laryngeal configuration at presentation, the cause of UVFP, and the type of HA for EIL were analyzed. Results: Ninety-four patients were included for analysis, with a mean follow-up period of 25.1 months (95% CI: 22.8–27.4 months). After primary HA injection, 22 patients (23.4%) underwent secondary procedures (rate: 13.1% per person-year), and most (63.6%) of the events occurred after one year from the first injection. The rate of secondary procedures within the first 12 months was 9.0% (14.1% and 4.3% for low-concentrated HA (LHA) and high-concentrated HA (HHA), respectively). The incidence of the secondary procedures was higher in the LHA group (18.2%) (*p* = 0.026) than in the HHA group (7.5%). Conclusions: The rate of secondary procedures was lower than the prediction based on the resorption time of HA, a finding that could be partly accounted for by both natural nerve recovery and a long-lasting effect of EIL. EIL with HHA had a lower rate of re-treatment than that with LHA, suggesting a better clinical utility for acute UVFP.

## 1. Introduction

Early injection laryngoplasty (EIL) with temporary materials is effective for UVFP and may reduce the need for permanent laryngoplasty in specific patient groups [1,2,3]. Since the introduction of distal-chip laryngoscopy, office-based EIL has become favored by UVFP patients [1,2,3,4,5] because it immediately adjusts the vocal position to improve the patients’ voice and swallowing functions [3,4,6,7,8,9]. The feasibility of office-based injection laryngoplasty (IL) and its excellent short- to mid-term voice outcomes have been demonstrated, as a majority of patients are satisfied with the correction of their breathy voice, aspiration, and chronic cough. However, its therapeutic effect might last for only a limited period of time, so some patients still have to have secondary procedures, such as repeated injections or framework surgeries. Thus, it is important to evaluate its long-term effect and the rate of additional treatments.

A randomized controlled trial with a 6-month follow-up performed by our group showed that EIL with hyaluronic acid (HA) improved mental health but could not enhance nerve regeneration [4]. In addition to the immediate voice effect of EILs using temporary materials, some authors have also considered their prolonged impact [2,3]. A recent systemic review addressing the question of whether EIL could reduce the need for permanent thyroplasty showed that, given the considerable heterogeneity among the studies, the long-term beneficial effect of EIL remains inconclusive [5]. Indeed, the variety of injection materials makes data synthesis difficult, and this motivated us to compare the therapeutic effects among different injection materials.

Over the years, various materials have been used for EILs, such as Gelfoam, carboxy-methylcellulose, calcium hydroxyapatite, autologous fat, and HA. HA is superior to other injectable materials because it has a lower risk of hypersensitivity reactions and is prone to remodeling scars [7,10,11]. Most patients with glottic insufficiency are satisfied with the immediate treatment effect from HA injection. However, the uncertainty of its longevity makes HA a less preferable material for intracordal injection. We previously reported the effect of EIL with Restylane [1]. The duration of the benefit of Juvéderm has also been reported for glottic insufficiency [12] with a mean duration of benefit of 10.6 months. However, the necessity for treatment not only relied on the objective measurements but also on the vocal demand. Vocal demand can differ according to individuals’ age, sex, occupation, and socioeconomic status. Thus, the decision for treatment or retreatment in acute UVFP can be partially subjective. To our knowledge, there is no literature focusing on the retreatment rate after the first EIL for UVFP and, most importantly, comparing the long-term effects between HA types. In this study, the primary aim was to determine the rate of additional injection laryngoplasty in acute UVFP patients after EIL with HA with at least one year of follow up. The secondary outcome was to identify the factors influencing their prognosis, such as HA type.

## 2. Materials and Methods

### 2.1. Study Design

**Figure 1:**
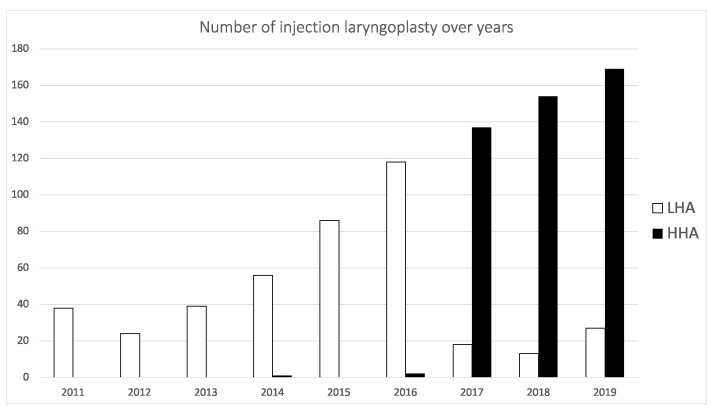
Number of injection laryngoplasties in the two HA groups over years. HHA = high-concentration HA group; LHA = low-concentration HA group.

The Institutional Review Board (IRB) of Chang Gung Memorial Hospital approved this study. It is a historical cohort study based in a tertiary medical center. Medical records from patients who underwent EIL with HA performed by a single laryngologist were examined from January 2015 to December 2018. In 2010, office-based IL with HA was introduced to our institute. The senior author (T.-J.F.) performed the first case in February 2011; a 22-year-old woman suffering from postviral UVFP received a Restylane injection 5 months after her hoarseness emerged. Thirty-seven patients received HA injection for glottic insufficiency in 2011, and the number increased to 202 in 2019 (Figure 1). The study time chosen was the period when the injection protocol was regarded as routine, but the HA materials were not limited to a certain type. The type of HA for each injection was not a subjective decision by the surgeon but rather based on the time point of injection. Before 2016, the Restylane family (Perlane or Restylane vital) was the only available HA for injection laryngoplasty because of the available published evidence [13]. Juvéderm Ultra Plus was introduced to our institute in 2016. According to the promising results shown in the report from Upton et al. [14], it quickly became the most common injectable material. Patients who met the inclusion criteria, acute unilateral vocal fold paralysis (UVFP) without spontaneous recovery diagnosed by videostroboscopy within 6 months, no history of previous laryngeal surgeries, and a follow up at least 12 months after the procedure were included in the analysis. A secondary procedure was performed if the patient had a subjective perception of deterioration in voice and requested further treatment. All patients received the HA injection on one side of the vocal fold.

### 2.2. Data Collection

The HA materials used in our practice include (1) Juvéderm Ultra Plus (24 mg/mL HA and 11% crosslinked) and (2) Restylane Vital and Perlane (20 mg/mL and 1% crosslinked forms) [12]. Based on their composition, Restylane Vital and Perlane were grouped as the Restylane family with a low concentration of HA (LHA group), whereas Juvéderm Ultra Plus was regarded as a high concentration of HA (HHA group). The etiology of UVFP was categorized as idiopathic, mass effect, iatrogenic, or thyroidectomy-related by reviewing the chart. The date of UVFP onset was defined as the date of symptom onset for nonsurgical-related UVFP or the operation date for iatrogenic cases. Preoperative videostroboscopy information was obtained, and each patient projected the/e/sound at their conversational pitch and intensity, during which time voice and vocal fold image movies were recorded by videolaryngostroboscopy. The recorded video was analyzed offline using ImageJ software (ImageJ 1.44; National Institutes of Health, Bethesda, MD, USA), which yielded the normalized glottal gap area (NGGA) by normalizing the area by the membranous vocal fold length using the equation developed by Omori et al. The glottal gaps were measured in both maximally open and maximally closed phases during vibration to yield open-phase and closed-phase NGGAs, respectively. In addition, for the magnitude of vibration, ΔNGGA was calculated as open-phase NGGA minus closed-phase NGGA [9].

### 2.3. Laboratory Voice Analysis

Patients were asked to read a standard message and pronounce vowels at a conversational pitch and loudness in a sound-insulated room. Their voices were recorded using a unidirectional dynamic microphone (Shure SM48; Shure Brothers Inc., Agua Prieta, Mexico) with a distance of 10 cm between the mouth and the microphone and an off-axis angle of 45°. Voices were sampled using voice-analysis software (Computerized Speech Lab Model 4300B, version 5.05; Kay Elemetrics Corp., Lincoln Park, NJ, USA), with a sampling rate of 25.6 kHz and 16-bit quantization. The modal fundamental frequency, perturbation of frequency (jitter) and amplitude (shimmer), and harmonic-to-noise ratio were tabulated from the recorded voice. Each parameter reflected a specific voice dimension. The values of jitter and shimmer reflected the deviation from voice periodicity and tended to increase in patients with voice problems. HNR quantified the amount of additive noise produced by turbulent glottal airflow and was suggested to be more analogous to the perception evaluation. The maximal phonation time represented the longest duration of sustaining a vowel/a/. The SZ ratio was the ratio of the voicing duration of /s/ to /z/, which represented the patient’s vocal control, with the ideal reference value being close to 1.0. Patients with UVFP tended to have a shorter maximal phonation time and a higher SZ ratio than healthy subjects [15].

### 2.4. Procedure of the HA Injection

The patients underwent the intracordal HA injection either in an office-based or an operating room setting administered by the senior author (T.-J.F.). Before injection, the nasal cavity was prepared by packing 1:100,000 2% lidocaine and epinephrine, and the oral cavity and oropharynx were anesthetized by gargling with 10% lidocaine. The patient sat upright with the neck extended. Under transnasal laryngoscopic guidance and visualization of the glottis on the monitor, around 0.5–1.0 mL 2% lidocaine was injected in the subcutaneous layer over the area of the cricothyroid (CT) space. For the hypersensitive patients, the intraluminal anesthesia was achieved through trans-tracheal or superior laryngeal nerve blocking. After local anesthesia, a needle was inserted at the level of the CT junction. After passing through the CT membrane, the needle was advanced upward and medially, and its tip was confirmed to be in the submucosal layer of the vocal cord by moving it carefully. Up to 1 mL HA materials were injected intracordally close to the medial aspect of the paraglottic space over the paralysis side until the glottal gap was closed completely. The surgical endpoint for LHA and HHA was the same, that is, to completely close the glottal gap when voicing during the procedure. To achieve such effect, the amounts for injection were customized. The patient was asked to project their voice during and at the end of the injection, an approach that could help confirm the vocal fold position and the effect to achieve a satisfactory voice [15].

### 2.5. Statistical Analysis

Statistical analysis was conducted using STATA (StataCorp LLC, College Station, TX, USA) version 15. Data are presented as the mean ± standard deviation (95% confidence interval) or number (percentage, %). The rate is expressed per person-year. To compare baseline characteristics between the participants with and without secondary procedures, Student’s *t*-test was used for continuous variables, and *X^2^* was employed for categorical variables. We also analyzed the baseline characteristics between the two HA groups. The time courses of the secondary procedures of the two HA groups were compared using the Kaplan–Meier method, and the difference was measured by the log-rank test. A *p* value <0.05 was regarded as statistically significant.

## 3. Results

### 3.1. Patient Characteristics

**Figure 2:**
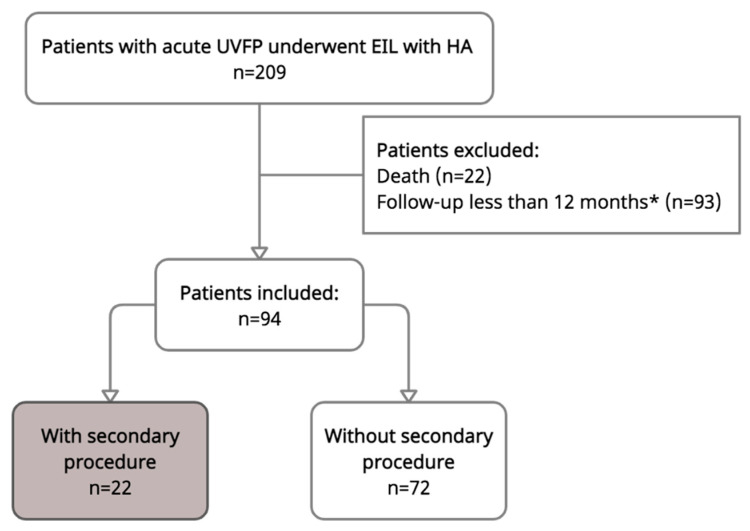
Flowchart illustrating inclusion/exclusion of the study population. EIL = early injection laryngoplasty. * By telephone consultation: patients with good voice (*n* = 37); patients with worsened voice (*n* = 9); not reachable (*n* = 47).

Two hundred and nine patients underwent injection laryngoplasty during the study period. One hundred and fifteen patients were excluded due to a follow up of less than 12 months, death, or spontaneous recovery of vocal motions at the end of follow up. Among those, by telephone consultation, it was found that 22 patients died, 47 were not reachable, and 37 of them stated they had a stable voice and swallowing conditions; only 9 of them had a worsened voice. Ultimately, a total of 94 patients met the inclusion criteria and were included in further analysis (Figure 2). Table 1 shows the distribution of baseline characteristics. The study population consisted of 50 men (53.2%) and 44 women (46.8%) with a mean age of 56.9 years (±SD: 1.5, CI: 53.9–59.9). Fifty-six (59.6%) patients had left side vocal paralysis. Seventy-nine (84.0%) of them had iatrogenic-related UVFP, and among them, 36 (38.3%) cases were caused by thyroidectomy. Half (50.0%) of the patients were injected with high-concentration HA (Juvéderm Ultra Plus), and the other half were injected with low-concentration HA (Restylane Vital, 42 (44.7%); Perlane, 5 (5.3%)). The average time from the onset of symptoms to treatment was 3.3 (±SD: 0.17, CI: 2.9–3.6) months, and the average follow-up time was 25.1 ± 1.2 (CI: 22.8–27.4) months.

### 3.2. Laryngeal Configuration and Voice Analysis

The results of the laryngeal configuration and voice analysis showed characteristics of acute UVFP. Specifically, all patients showed a wide closed-phase NGGA (10.2, SD: 1.1, 95% CI: 8.0–12.4) and ∆ open-closure NGGA (7.4, SD: 0.47, CI: 5.9–9.0). They all had a short maximal phonation time (4.5 s, SD: 0.53, CI: 3.8–5.3), a lower level of harmonic-to-noise ratio (6.1, SD: 0.32, CI: 5.5–6.8), and higher values of SZ ratio (2.2, SD: 0.07, CI: 2.0–2.5), shimmer (0.9, SD: 0.03, CI: 0.7–1.0), and jitter (5.3, SD: 0.19, CI: 4.4–6.1) [16]. One month after EIL with HA, both the open and closed NGGA were reduced significantly. Furthermore, in patients who did not receive secondary procedures, the NGGA at the last follow up did not change with time in comparison to the NGGA immediately after EIL, a finding implying that those without secondary procedures had durable effects induced by their first EIL (Table 2).

### 3.3. Secondary Procedure after EIL with HA

A secondary procedure, including a second HA injection or permanent laryngoplasty, was counted as a failure event. After at least 12 months of follow-up, 22 of the patients underwent secondary procedures (rate: 13.1%) during the follow-up period. Seventeen of them received a second HA injection, and five received permanent laryngoplasty. Two patients underwent HA injection more than twice because of specific general health concerns. One patient had a mediastinal surgery history and had IL three times: 1.6 months after the injury with LHA, 5.1 months after the injury with HHA, and 16.8 months after the injury with HHA. The other patient with a history of esophageal surgery received four IL procedures: 11.4 months after the injury with LHA, 14.4 months after the injury with LHA, 18.6 months after the injury with LHA, and 47.1 months after the injury with HHA. Both of them had a stable voice after multiple injections.

From the comparisons of baseline characteristics between patients with and without secondary procedures, the HA material used in the primary injection was closely associated with the possibility of retreatment (*p* = 0.015) during the follow-up period (Table 1). People injected initially in the LHA group accounted for the majority (72.7%) of the second injection events. There were no differences between patients with and without secondary procedures in terms of sex, age, paralysis side, etiology of UVFP, time to treatment, preoperative laryngeal configuration, or preoperative voice analysis.

Moreover, 8 out of 22 patients received their secondary procedures within one year after their first HA injection. The overall rate of secondary procedures within the first 12 months was 9.0% (14.1% and 4.3% for LHA and HHA, respectively). Overall, among those who underwent secondary procedures, 6 (incidence rate: 7.5%) and 16 (incidence rate: 18.2%) patients received HHA and LHA, respectively, for their first injection (Table 3).

### 3.4. Comparable Patient Characteristics between the HHA and LHA Groups

Table 4 shows the baseline characteristics between the HHA and LHA groups. There were no differences in age, sex, side of palsy, time to treatment, cause of nerve injuries, or laryngeal configuration. The LHA group had a longer follow-up time (27.8 months vs. 22.4 months, *p*-value: 0.02), whereas the HHA group had worse jitter (HHA vs. LHA: 6.41 vs. 4.41, *p*-value: 0.017) and shimmer (1.04 vs. 0.75, *p*-value: 0.026) values.

### 3.5. Survival Analysis of the Primary HA Injection

**Figure 3:**
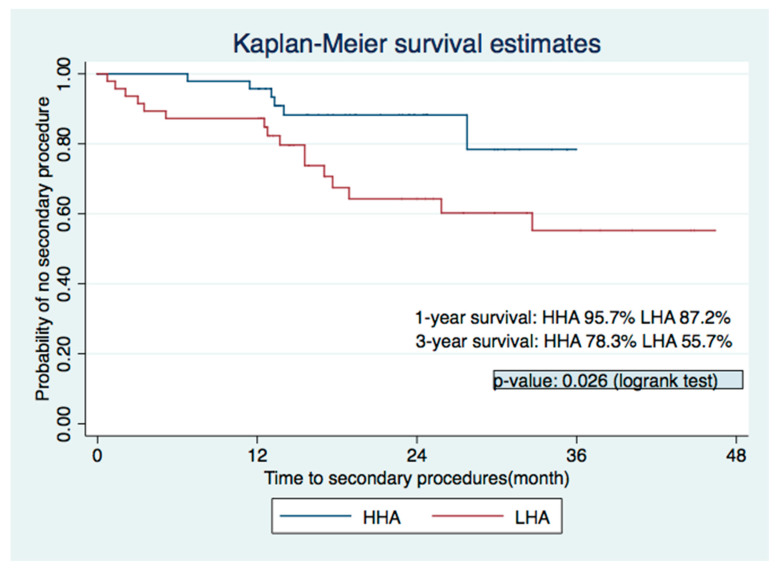
Kaplan–Meier estimates showed significantly better prognosis in the HHA group. HHA = high-concentration HA group; LHA = low-concentration HA group.

Figure 3 shows the results of the Kaplan-Meier survival analysis that compared the persistence of the primary HA injection’s effect across the two different concentration groups. Compared with the HHA group, the LHA group had a lower persistence rate, as the one-year rate without secondary procedures was 95.7% in the HHA group and 87.2% in the LHA group. The difference increased over the years. Three years after the procedure, the probability of no secondary procedure was 78.3% in the HHA group and 55.7% in the LHA group (*p*-value = 0.026, log-ranked test).

## 4. Discussion

HA can form a transient polymer network system whenever shear force is applied. HA can be transformed and autonomously reassembled when the force is removed [7,17], a physical property that has made HA an ideal dermal filler for decades. A recent study indicated that HA had a better residence time, injection localization, and tissue compatibility than carboxymethylcellulose gel, one of the most common materials used for IL [18]. Although HA has not been approved by the U.S. Food and Drug Administration (FDA) for vocal injection, robust evidence shows its effectiveness and safety [4,9,11,12,15,19,20]; thus, HA was recently accepted as an injectable material for vocal fold augmentation.

Our institute started to provide an office-based IL procedure with HA in February 2011, and the number of patients undergoing the procedure has increased annually (Figure 1). During the initial stage of our practice, the Restylane family (Restylane and Perlane), which had published evidence for intracordal injection [9,21,22,23,24], was the only available material for injection. Since 2016, after its safety was proven by Upton et al. [14], Juvéderm Ultra Plus was also provided in our service when its immediate treatment effect was noted to be equivalent to that of the Restylane family. Because of its lower expense in our institute, most EIL procedures have been performed using Juvéderm Ultra Plus since then. Thus, the selection of the materials was solely made based on the time when we performed the procedure. Comparable patient characteristics between the two groups further proved that there was no selection bias in the present cohort.

The injection materials for IL have different characteristics [7,9,14,22,24,25,26], which are closely related to the persistence of the treatment effect. HA materials vary widely in their particle size, percentage of crosslinked form, and concentration of HA present, yielding different physical characteristics [27]. The Restylane family (such as Restylane Vital and Perlane) are biphasic, particle-formed HA gels, whereas Juvéderm Ultra Plus is a kind of monophasic, homogeneous-formed HA. Juvéderm Ultra Plus consists of a higher degree of crosslinked form and a higher HA concentration than Resylane and Perlane [7,17,27]. The concentration affects the longevity of injection materials and further influences the persistence of the treatment effect [7,27]. However, there is no literature comparing the effect of different concentrations of HA with the treatment effect of phonosurgery. The present study showed that after an average follow up of 25.1 months, more than 75% of acute UVFP patients had adequate vocal effects without the need for further treatment after a single HA injection. The type of injection material is the only factor that influences the need for secondary procedures. We propose that high-concentration HA preparation (Juvéderm Ultra Plus) might last longer in the vocal fold and, thus, prolong the treatment effect of EIL.

**Figure 4:**
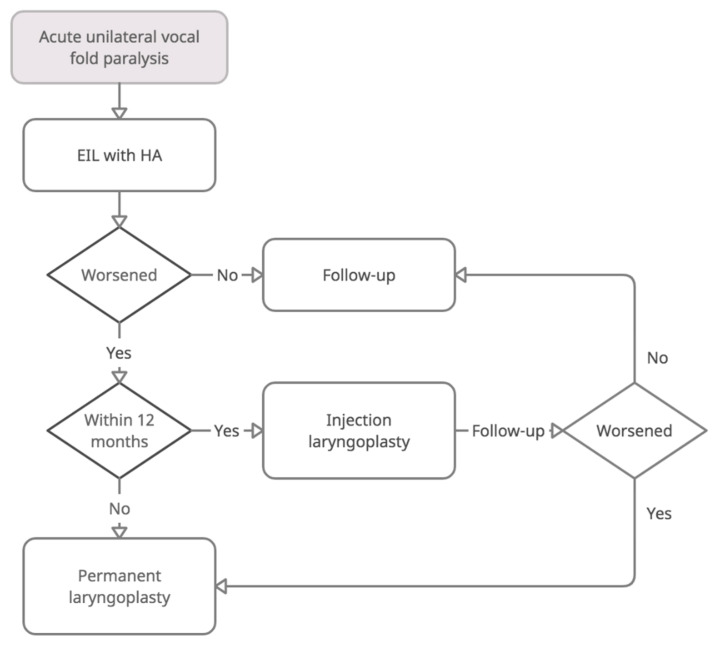
Recommendation for treatment guidelines for acute unilateral vocal fold paralysis. EIL = early injection laryngoplasty; HA = hyaluronic acid.

For UVFP patients who intend to receive EIL, we noticed that the greatest concerns were “How long can I be well?” and “Do I need to receive further treatments?”. Individuals may hesitate to accept EIL with HA because of the uncertainty of its longevity. From our previous randomized controlled trials, we found that although HA may be degraded within weeks, the related vocal effect may last [4]. The results of this study showed that approximately 93% of people with acute UVFP felt well and did not need secondary procedures within 12 months. Due to the low morbidity rate, EIL with HA should be encouraged, and repeated office-based IL could also be an alternative before performing permanent thyroplasty. In the present cohort, 15 patients received HA injection twice, 1 patient had three sessions, and another had four sessions of HA injection. The last two cases did not proceed to permanent laryngoplasty because of relatively poor general health status. Only five patients (5.3%) underwent permanent laryngoplasty at the end of follow up, ranging from 10 to 19 months from the onset of dysphonia. The interval between primary HA EIL and permanent laryngoplasty was 12 months (5.1–17 months). According to these results, we suggest a repeated HA injection for early deterioration within 12 months and permanent laryngoplasty for late deterioration (Figure 4), which is helpful for patient consultation before EIL.

Some may doubt that patients might refuse further treatment with a worsened voice quality. The requirement of further laryngoplasty may be different based on patient age, vocal demand, and socioeconomic status. However, we noted that the follow-up glottal gap in patients without further treatment remained small, which suggested that their voice did not change much with time. According to the consistency of the follow-up data, we proposed that a low secondary procedure and permanent laryngoplasty rate could be expected from a single session of EIL with HA, especially when using a high-concentration form.

### Study Limitations

There are several limitations of this study. First, the need for secondary injections might have been influenced by the patients’ subjective needs. The reason for and timing of decisions differed across patients. Second, the results might be changed over time and the rate of secondary procedures needs to be confirmed by a longer follow up. Third, the present study was a historical cohort review. Although there was no evidence of selection bias between the two groups, we believe that a more robust conclusion could only be achieved by a randomized controlled trial.

## 5. Conclusions

EIL with HA is a safe and effective treatment for acute UVFP. The effect of augmentation using HA is durable and satisfactory. It lasts longer than the duration predicted based on the resorption time of HA. Furthermore, the concentration of HA influences the sustainability of the treatment effect. From the point of retreatment incidence, HHA had a better clinical utility for treating acute UVFP.

## Figures and Tables

**Table 1:** Patient demographics and comparisons between patients with and without secondary procedures (*n* = 94).

		*n* (%) or Mean (95% CI)	Secondary Procedures	None	*p*-Value
		*n* = 94	*n* = 22	*n* = 72	
Age		56.9 (53.9–59.9)	51.6 (43.9–59.4)	58.5 (55.3–61.6)	0.055
Gender	Male	50 (53.2)	12 (24.0)	38 (76.0)	0.9
	Female	44 (46.8)	10 (22.7)	34 (77.3)	
Palsy side	Left	56 (59.6)	14 (25.0)	42 (75.0)	0.7
	Right	38 (40.4)	8 (21.1)	30 (79.0)	
Initial injection material	HHA	47 (50.0)	6 (12.8)	41 (87.2)	0.015
	LHA	47 (50.0)	16 (34.0)	31 (66.0)	
Time to treatment (months)		3.27 (2.93–3.60)	3.41 (2.43–4.39)	3.23 (2.89–3.57)	0.65
Follow-up time (months)		25.1 (22.8–27.4)	27.5 (23.0–31.9)	24.4 (21.7–27.1)	0.27
Etiology	Idiopathic	8 (8.5)	2 (25.0)	6 (75.0)	0.77
	Mass effect	7 (7.5)	1 (14.3)	6 (85.7)	
	Iatrogenic	43 (45.7)	12 (27.9)	31 (72.1)	
	Thyroidectomy	36 (38.3)	7 (19.4)	29 (80.6)	
Laryngeal configuration (*n* = 89)			*n* = 22	*n* = 67	
Closed-phase NGGA		10.2 (8.0–12.4)	7.5 (5.5–9.6)	11.1 (8.3–13.9)	0.2
Open-phase NGGA		18.7 (15.5–21.9)	17.5 (7.1–27.8)	19.1 (16.3–22.0)	0.7
∆ Open-closure NGGA		7.4 (5.9–9.0)	5.5 (3.0–8.2)	8.0 (6.2–9.9)	0.2
Voice analysis (*n* = 75)			*n* = 21	*n* = 54	
Maximum phonation time (s)		4.5 (3.8–5.3)	4.2 (3.1–5.2)	4.7 (3.7–5.6)	0.5
SZ ratio		2.2 (2.0–2.5)	2.0 (1.6–2.4)	2.3 (2.0–2.6)	0.2
Fundamental frequency (Hz)		183.0 (168.9–197.0)	191.0 (157.6–224.4)	179.8 (164.6–195.1)	0.5
Jitter (%)		5.3 (4.4–6.1)	5.3 (3.6–6.9)	5.3 (4.3–6.3)	0.9
Shimmer (dB)		0.9 (0.7–1.0)	0.7 (0.5–0.9)	0.9 (0.8–1.1)	0.1
Harmonic-to-noise ratio		6.1 (5.5–6.8)	6.5 (5.1–7.9)	6.0 (5.2–6.8)	0.5

HHA = high-concentration HA group; LHA = low-concentration HA group; NGGA = normalized glottic gap area.

**Table 2:** Difference of laryngeal configuration before and one month after the EIL; difference of laryngeal configuration before the EIL and at last follow-up in patients without secondary procedures.

	Preoperative (*n* = 89)	1 Month (*n* = 82)	*p*-Value	Without Second Procedure (*n* = 70)	*p*-Value *
Closed-phase NGGA	10.2 (8.0–12.4)	1.9 (1.2–2.6)	<0.001	0.9 (0.5–1.2)	<0.001
Open-phase NGGA	18.7 (15.5–21.9)	9.2 (8.2–10.1)	<0.001	11.0 (9.6–12.5)	<0.001
∆ Open-closure NGGA	7.4 (5.9–9.0)	7.3 (6.3–8.2)	0.47	10.1 (8.7–11.6)	0.0067

EIL = early injection laryngoplasty; NGGA = normalized glottic gap area; *p*-value *: paired *t* test comparing laryngeal configuration before the EIL and at last follow-up in patients without secondary procedures.

**Table 3:** Incidence rate of secondary procedures between the two HA groups.

Material	<1 Year (Rate)	1–2 Year (Rate)	>2 Year (Rate)	Events	Rate (Person-Year%)
HHA group	2 (4.3%)	3 (11.0%)	1 (16.6%)	6	7.5%
LHA group	6 (14.1%)	8 (34.0%)	2 (9.1%)	16	18.2%
Rate (person-year %)	8 (9.0%)	11 (21.6%)	3 (10.7%)	22	13.1%
Percentage (%)	8 (36.4%)	11 (50.0%)	3 (13.6%)	22	

HHA = high-concentration HA group; LHA = low-concentration HA group.

**Table 4:** Patient demographics between HA groups (*n* = 94).

		HHA *n* (%) or Mean (95% CI)	LHA *n* (%) or Mean (95% CI)	*p*-Value
		*n* = 47	*n* = 47	
Age		57.5 (53.8–62.3)	56.2 (51.4–61.0)	0.66
Gender	Male	23 (48.9%)	27 (57.5%)	0.41
	Female	24 (51.1%)	20 (42.6%)	
Side	Left	26 (55.3%)	30 (63.8%)	0.40
	Right	21 (44.7%)	17 (36.2%)	
Time to treatment (months)		3.36 (2.96–3.77)	3.17 (2.62–3.72)	0.58
Follow-up time (months)		22.40 (20.17–24.63)	27.82 (23.81–31.83)	0.020
Etiology	Idiopathic	2 (4.3%)	6 (12.8%)	0.11
Mass effect		4 (8.5%)	3 (6.4%)	
Iatrogenic		18 (38.3%)	25 (53.2%)	
Thyroidectomy		23 (48.9%)	13 (27.7%)	
Laryngeal configuration		*n* = 45	*n* = 44	
Closed-phase NGGA		9.86 (7.08–12.65)	10.56 (7.03–14.09)	0.75
Open-phase NGGA		19.90 (14.70–25.10)	17.52 (13.59–21.45)	0.46
∆ Open-closure NGGA		7.90 (5.79–10.00)	6.96 (4.62–9.29)	0.55
Voice analysis (*n* = 75)		*n* = 32	*n* = 43	
Maximum phonation time (s)		4.37 (31.4–5.61)	4.63 (3.65–5.60)	0.74
SZ ratio		2.46 (2.05–2.86)	2.07 (1.81–2.32)	0.087
Fundamental frequency (Hz)		181.24 (157.69–204.80)	184.23 (166.27–202.19)	0.84
Jitter (%)		6.41 (4.93–7.89)	4.41 (3.50–5.32)	0.017
Shimmer (dB)		1.04 (0.82–1.28)	0.75 (0.59–0.91)	0.026
Harmonic-to-noise ratio		5.37 (4.37–6.38)	6.70 (5.78–7.62)	0.054

HHA = high-concentration HA group; LHA = low-concentration HA group; NGGA = normalized glottic gap area.
